# Determinants of tRNA Recognition by the Radical SAM Enzyme RlmN

**DOI:** 10.1371/journal.pone.0167298

**Published:** 2016-11-30

**Authors:** Christina M. Fitzsimmons, Danica Galonić Fujimori

**Affiliations:** 1 Chemistry and Chemical Biology Graduate Program, University of California San Francisco, San Francisco, California, United States of America; 2 Department of Cellular and Molecular Pharmacology, University of California San Francisco, San Francisco, California, United States of America; 3 Department of Pharmaceutical Chemistry, University of California San Francisco, San Francisco, California, United States of America; Keio University, JAPAN

## Abstract

RlmN, a bacterial radical SAM methylating enzyme, has the unusual ability to modify two distinct types of RNA: 23S rRNA and tRNA. In rRNA, RlmN installs a methyl group at the C2 position of A2503 of 23S rRNA, while in tRNA the modification occurs at nucleotide A37, immediately adjacent to the anticodon triplet. Intriguingly, only a subset of tRNAs that contain an adenosine at position 37 are substrates for RlmN, suggesting that the enzyme carefully probes the highly conserved tRNA fold and sequence features to identify its targets. Over the past several years, multiple studies have addressed rRNA modification by RlmN, while relatively few investigations have focused on the ability of this enzyme to modify tRNAs. In this study, we utilized *in vitro* transcribed tRNAs as model substrates to interrogate RNA recognition by RlmN. Using chimeras and point mutations, we probed how the structure and sequence of RNA influences methylation, identifying position 38 of tRNAs as a critical determinant of substrate recognition. We further demonstrate that, analogous to previous mechanistic studies with fragments of 23S rRNA, tRNA methylation requirements are consistent with radical SAM reactivity. Together, our findings provide detailed insight into tRNA recognition by a radical SAM methylating enzyme.

## Introduction

**Fig 1:**
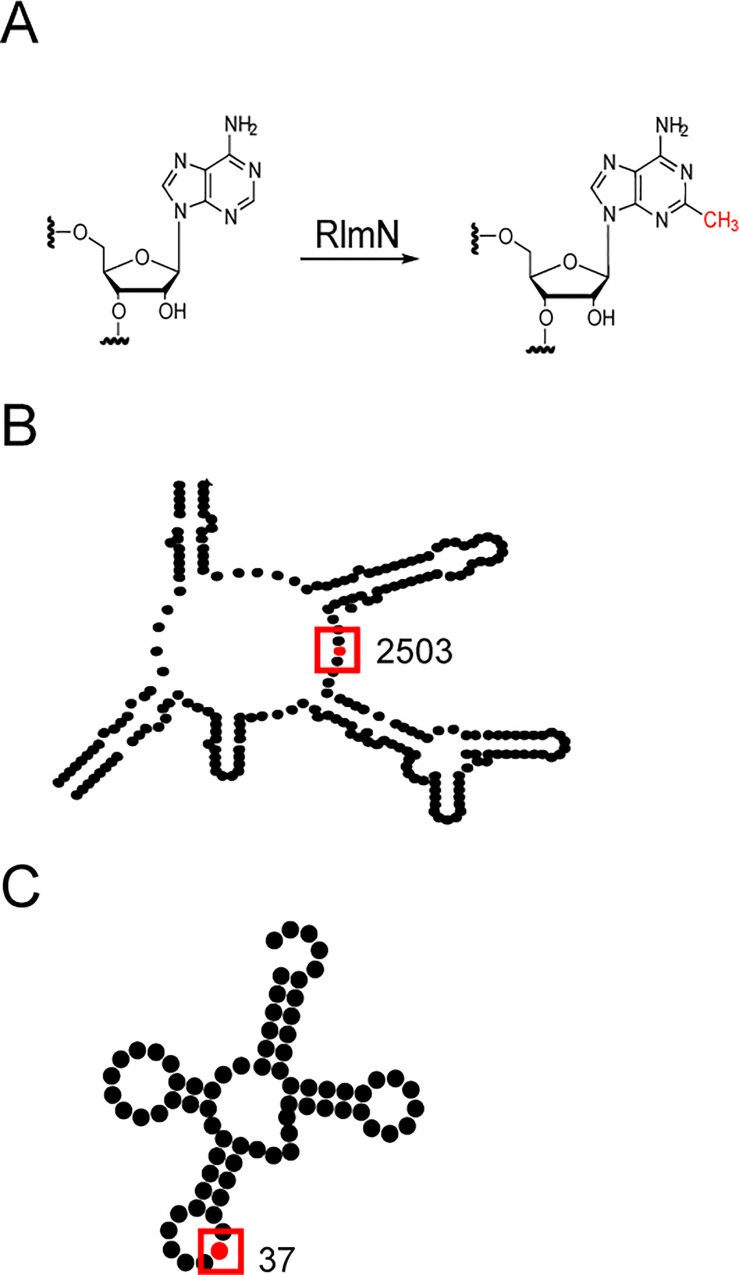
RNA methylation catalyzed by RlmN. (A) RlmN catalyzes the conversion of adenosine (A) to 2-methyladenosine (m^2^A). RlmN modifies position A2503 in 23S rRNA (B) and position A37 in select tRNAs (C).

Methylation of RNA on the four canonical bases modulates its structure and function [1], allowing it to fulfill its numerous roles in the cell. These modifications are introduced by RNA methyltransferases (MTases). To accomplish these transformations, RNA MTases utilize several general mechanisms. The exocyclic nitrogens in the nucleobases [2–4] as well as oxygen atoms at the 2' position of the ribose [5,6] are nucleophilic sites. MTases promote methylation at these sites through favorable orientation of the RNA and the methyl group donor. Unlike methylation of nitrogen or oxygen atoms, methylation of cytosine or uridine at the C5 position requires a different mechanism, because the target position is not nucleophilic. Covalent catalysis via a Michael addition activates the C5 carbon and accounts for methylation at these sites [7–16]. In contrast, methylation at the unreactive C2 and C8 positions of adenosines requires a different enzymatic mechanism and is catalyzed by members of the radical *S*-adenosyl-l-methionine (SAM) superfamily (Fig 1A) [17,18].

Studies on RlmN and Cfr, two bacterial radical SAM methylating enzymes, have established key mechanistic features of radical SAM methylation of RNA. A unique feature of these enzymes is their ability to utilize both homolytic and heterolytic reactivity of SAM to carry out methylation of the C2 and C8 amidine carbons of adenosine [19–21]. The first equivalent of SAM is used to methylate a conserved cysteine residue (C355), unassociated with the four iron-four sulfur ([4Fe-4S]) cluster, to form a protein-bound methyl thioether [19,22–24]. A second equivalent of SAM, coordinated by the [4Fe-4S] cluster in these proteins, is then cleaved homolytically to generate a 5′-deoxyadenosyl radical (5′-dA•), a canonical feature of radical SAM catalysis [25]. This highly reactive radical species then abstracts a hydrogen atom from the premethylated cysteine 355 to form a thiomethylene radical. The methylene radical then adds into the substrate carbon to form a covalent RNA-protein adduct, which has been trapped by mutagenesis [22] and characterized spectroscopically [23,24]. A second conserved cysteine residue resolves the covalent RNA-protein intermediate, forming the methylated product.

In addition to its mechanism of action, RlmN is also unusual in that it is able to act on both rRNA and tRNA (Fig 1B and 1C) [26]. RNA modifying enzymes are generally highly specific for the type of RNA that they modify (*e*.*g*. tRNA, rRNA, mRNA, etc). To date, there are only two other enzymes in bacteria, the pseudouridine synthase RluA and the methyltransferase TrmA, that have been shown to modify multiple types of RNA [7–9,27–31]. When acting on rRNA, RlmN methylates the C2 position of A2503 in 23S rRNA. While the biological role of this modification is not fully understood, it has been proposed to contribute to translational fidelity [26,32,33]. Lack of methylation of A2503 has a minimal effect on the cell fitness and has been shown to slightly increase linezolid resistance in *Staphylococcus aureus* [34–36]. Additionally, absence of the C2 methylation of A2503 can also cause resistance to the pleuromutilin antibiotic tiamulin, as demonstrated for catalytically inactive RlmN variants obtained by directed evolution [37]. When acting on tRNA, RlmN methylates the C2 position of A37, a nucleotide located in the anticodon stem loop (ACSL) immediately adjacent to anticodon triplet [26,38,39]. While the functional significance of A37 methylation is poorly understood, it is interesting to note that RlmN only modifies a subset of tRNAs that contain an A at position 37.

Structures of the RlmN C118A mutant in complex with one of its tRNA substrates, tRNA^Glu^_UUC_, have recently been reported, shedding light on the structural aspects of tRNA recognition by this enzyme [40]. These structures, obtained by trapping of the covalent enzyme-substrate complex through protein mutagenesis, indicate that RlmN recognizes the overall shape of the co-crystallized tRNA^Glu^_UUC_ through interaction with the sugar-phosphate backbone of the D-arm and the 3' acceptor end of the tRNA. Additionally, the study revealed that the most extensive protein-tRNA interactions are located in the ACSL region, which is remodeled by the enzyme to allow access to the substrate nucleotide.

Intrigued by the ability of RlmN to modify both rRNA and tRNA, we sought to determine the tRNA features necessary for methylation by RlmN. In addition, the compact, well-defined structure of tRNA combined with its short length (approx. 75 nt) relative to 23S rRNA (2904 nt), provides an excellent framework to investigate RNA recognition by RlmN. To understand why RlmN methylates only a subset of tRNAs that have an adenosine at position 37, we prepared chimeric RNA constructs that combine features of substrate and non-substrate tRNAs and assessed their ability to serve as RlmN substrates. In addition, we generated ACSL point mutants to investigate how the nature of the nucleotides in this critical recognition region impacts RlmN-mediated methylation. Our results indicate that the local sequence of the ACSL plays a critical role in the ability of RlmN to discriminate and modify tRNA substrates.

## Materials and Methods

### General Methods

Anaerobic manipulations were carried out in a glovebox (MBraun) under a 99.997% nitrogen atmosphere containing less than 2 ppm oxygen. All reagents were analytical grade or the highest grade commercially available and used as supplied unless noted otherwise. Plasmids containing *Escherichia coli* K-12 tRNA genes were synthesized by Gene Oracle. [^14^C-*methyl*]-SAM was purchased from Perkin Elmer. Oligonucleotide PCR primers and ACSL 17-mer RNA were synthesized by Integrated DNA Technologies (IDT). PCR purification was performed with Qiagen PCR purification kits and protocol. Enzymes for *in vitro* transcription were purchased from New England Biolabs (NEB) unless otherwise noted. The tRNA used in *in vitro* methylation assays was purified using Zymo Research RNA Clean & Concentrator kit unless otherwise noted. Statistics and graphical analysis were performed in GraphPad Prism VI.

### Expression, Purification and Reconstitution of WT and Mutant RlmN Proteins

Wild-Type (WT) RlmN and RlmN prepared without the [4Fe-4S] cluster (ApoRlmN) were prepared by modified versions of previously published protocols [19,20]. Briefly, enzymes were over expressed in M9 minimal media supplemented with 75 μM 1,10-phenanthroline. Enzymes were purified by Talon chromatography. Following Talon purification, the iron-sulfur cluster in WT RlmN was reconstituted by chemical reconstitution and further purification by FPLC on a MonoQ 10/100 GL column (GE Healthcare Life Sciences) using a linear gradient of buffer A (50 mM Tris-HCl, pH 8.0, 10% glycerol, 50 mM NaCl) and buffer B (50 mM Tris-HCl, pH 8.0, 10% glycerol, 1 M NaCl). Following Talon purification, ApoRlmN was immediately purified by FPLC on the MonoQ column as described above. Mutant C118A RlmN was overexpressed, purified by Talon affinity chromatography, and reconstituted as described previously [20,22]. Following chemical reconstitution, anion exchange chromatography employing either HiTrap Q HP or MonoQ columns was performed and fractions containing protein were combined and concentrated for storage at -80°C. Protein concentration was determined by the Bradford method.

### Expression and Purification of Flavodoxin and Ferredoxin:NADPH Oxidoreductase

Flavodoxin (FldA) and Ferredoxin:NADPH Oxidoreductase (Fpr) were expressed and purified as described previously [22,41]. Briefly, pETARA expression vectors containing an N-terminal GST fusion incorporating a Tobacco Etch Virus (TEV) protease cleavable linker N-terminal to FldA and Fpr were transformed into BL21 (DE3) *E*. *coli*. Overnight cultures were grown in LB media containing 100 μg/mL ampicillin and used to inoculate 1L expression cultures. Cells were grown to OD_600_ of 0.6 with shaking at 37°C. Isopropyl β-d-1-thiogalactophyranoside (IPTG) was added to a final concentration of 200 μM. Incubation and shaking continued for another 4 hours, after which the cells were harvested and the pellets stored at -80C. Cells were resuspended in lysis buffer (50 mM HEPES pH 7.5, 150 mM NaCl, 1 mM dithiothreitol (DTT), 1 mM phenylmethylsulfonyl fluoride (PMSF)) with the addition of 1 mg/mL lysozyme. The resuspended cells were treated with lysozyme for 1 hr at room temperature, after which the cells were lysed by sonication on ice. Cellular debris was removed by ultracentrifugation and the clarified lysate was incubated with 2 mL of glutathione sepharose 4B beads (GE Healthcare) at 4°C with rocking for 1 hour. The protein bound resin was washed with 10 bed volumes of wash buffer (50 mM HEPES, pH 8.0, 10% (v/v) glycerol, 1 mM DTT) and resuspended in the same buffer. TEV protease was added to 1% (w/w) of protein. Incubation at 4°C with rocking was continued overnight. Following cleavage, the resin was washed with the wash buffer and the eluted cleaved proteins were collected, concentrated, and stored at -80°C.

### Alignment of tRNA Substrates and Non-substrates

*E*. *coli* DNA sequences for known *in vivo* substrates (“substrates”) and tRNAs containing adenosine at position 37 that is not known to be modified (“non-substrates”) were extracted from the MODOMICS database [42–44]. Alignment of substrate and non-substrate tRNAs was performed by T-Coffee alignment program within the Jalview platform. Alignment visualization and statistics were performed in Jalview (S1 Fig) [45].

### Construction and Purification of WT and Point Mutant tRNAs

Plasmids containing the tRNA gene sequences were synthesized by Gene Oracle with the following general construction: 5'-T7 promoter sequence-tRNA gene-Bam-HI restriction site-3'. In both the tRNA^Gln^_UUG_ and tRNA^Gln^_CUG_ constructs, the U at position 1 was removed, so that these genes begin at position 2. For the His, Asp, and Glu genes, an additional G was added 5' of position 1. These changes were made because a G is required at positions 1 and 2 for transcription by T7 RNA polymerase [46–48].

No modifications were made to the sequences for Asp or Gly. Point mutations were introduced to WT tRNA using site-directed mutagenesis. Plasmids were linearized by overnight digest at 37°C with BamHI-HF (NEB) and purified for use in *in vitro* transcription reactions.

#### Preparation of rRNA Substrates

Segments of the 23S rRNA gene were amplified using the plasmid pKK3535 as a template [20]. The primers used for PCR amplifications were F-2496: 5′-GGGCACCTCGA-3' and R-2582: 5'-CCA GCT CGC GTA CCA CTT TAA A-3'. The forward PCR primer also included the T7 RNA polymerase promoter sequence 5'-TAATACGACTCACTATAGG-3'. PCR products were purified using the Qiagen PCR purification kit and subsequently used for *in vitro* transcription.

#### Preparation of Chimeric tRNA Substrates

Chimeric tRNAs were constructed by overlap extension PCR. The first of two PCRs created a linear tRNA gene. The sequences for tRNA^Gln^_UUG_ and tRNA^Gly^_CCC_ were selected as the substrate and non-substrate sequences, respectively. The sequences of the final chimeric tRNAs are listed in S1 Table. Overlap extension primers were designed to be approximately 45 bases long and contain a 10–12 base overlap. PCR was performed with Phusion DNA polymerase in the accompanying Phusion GC buffer supplemented with 50 mM KCl and 2% betaine. For each 50 μL reaction, 400 pmol of each primer was used as template.

PCR products were gel-purified and then subjected to a second round of PCR to amplify the gene of interest. All forward PCR primers for this round included the T7 RNA polymerase promoter sequence 5'-TAATACGACTCACTATA-3', followed by several nucleotides corresponding to the specific tRNA. PCR was performed with Phusion DNA polymerase in the accompanying GC buffer, supplemented with 2% DMSO and 2% betaine. Purity was ensured by acrylamide gel electrophoresis before beginning *in vitro* transcription.

#### *In vitro* Transcription of tRNAs

RNAs were produced by T7 RNA polymerase *in vitro* transcription from linearized plasmid or PCR amplicon templates in 1 mL volume following standard protocols [47]. Reactions contained 40 mM HEPES, pH 8.1, 2 mM Spermidine, 0.01% Triton X-100, 50 mg/mL PEG 8000, 20 mM DTT, 8 mM rGTP, pH 8.0, 5 mM rUTP, pH 8.0, 5 mM rATP, pH 8.0, 5 mM rCTP, pH 8.0, 30 mM MgCl_2_, and 4 U Thermostable Inorganic Pyrophosphatase (TIPP) (NEB). Transcription reactions were incubated at 37°C for 6 hours and subsequently quenched with 100 μL of 0.5 M EDTA, pH 8.0. The RNAs were purified by 8% Polyacrylamide / 8 M Urea gel and the bands were excised by UV-shadowing. Gel-excised RNA was extracted in nuclease-free water and precipitated overnight at -20°C with 0.3 M sodium acetate, pH 5.2 and ethanol. Precipitated RNAs were washed with 70% ethanol and the pellet was air-dried before resuspension in nuclease-free water.

#### RNA Folding and Native Gel Analysis of tRNA

The tRNAs were folded on a thermocycler in a buffer containing 100 mM KCl. First, the tRNAs were heated to 95°C for 3 min, and then cooled slowly to 65°C, at which point MgCl_2_ was added to a final concentration of 5 mM. The temperature was then lowered over the course of 60 minutes to 4°C. Uniform RNA folding was determined by 0.5x TBE, 8% polyacrylamide native gel stained with Sybr Gold and visualized on a BioRad ChemiDoc MP Imaging System (BioRad) (S2 Fig).

#### Determination of tRNA Melting Temperature

PIPES (50 mM, pH 7.5) and (NH_4_)_2_SO_4_ (30 mM) were added to tRNAs dissolved in distilled water [46]. The changes in absorbance with increasing temperature were measured using a Cary-100 UV Vis Spectrophotometer coupled with a 6 x 6 multicell holder with temperature control. The temperature was increased from 20°C to 95°C at a rate of 0.1°C/min. The tRNA samples were allowed to equilibrate at 20°C for 10–15 minutes before thermal denaturation profiles were obtained. Absorbance was monitored at 260 nm. RNA samples were measured in a quartz spectrophotometer cell with a tight septum cap (Starna Cells). A computer generated plot was measured for each profile and the melting temperature (Tm) was calculated as the inflection point (second derivative d^2^A/dT^2^) of absorbance with respect to temperature. All measurements were performed in triplicate (S2 Table). *In silico* secondary structure predictions were made by the Vienna RNA Websuite RNAFold Program [49].

### Activity Assay

Radioactive methylation reactions were performed similar to previously described conditions [50]. Briefly, reactions contained 10 mM MgCl_2_, 2mM DTT, 20 μM FldA, 2 μM Fpr, 2 μM RNA, 1 μM RlmN, and 30.7 μM (0.075 μCi) [^14^C-*methyl*]-SAM (Perkin Elmer) (58 mCi/mmol) and 1 mM NADPH in 100 μL reaction buffer (100 mM HEPES, pH 8.0, 100 mM KCl). All reaction components except the radiolabeled SAM and NADPH were made anaerobic by purging with argon prior to mixing in an MBraun glovebox. The reaction mixture was then removed from the anaerobic chamber in a gas-tight glass vial. [^14^C-*methyl*]-SAM was aliquoted in a second gas-tight vial outside of the glovebox and dried by an argon stream. To this vial, which was kept under argon pressure, the reaction mixture was added using a gas-tight syringe. The vial was allowed to pre-incubate at 37°C for 5 min. The reaction was initiated by addition of anaerobic NADPH and incubated at 37°C for 60 min. Immediately after initiation of the reaction, a 5 μL aliquot was removed. This sample was used to determine the total amount of radioactivity in the vial. After 1 hour, reactions were quenched by addition of cold SAM to a final concentration of 120 μM. RNA was then purified from the reaction mixture using the RNA Clean & Concentrator Kit (Zymo Research). Recovered RNA was added to vials containing Ultima Gold scintillation fluid. The amount of incorporated radioactivity in the products was measured using a Beckman-Coulter multipurpose scintillation counter (Fullerton, CA). All reactions were performed in duplicate at minimum. The experimentally measured radioactivity in the product was normalized to a theoretical maximum (100% methylation). The normalized results were then plotted in GraphPad Prism VI.

### HPLC Separation and Identification of Methylated Adenosine

Methylated RNA was purified using the RNA Clean & Concentrator Kit (Zymo Research). The purified RNA was subsequently enzymatically digested to individual mononucleosides using nuclease P1 (Sigma Aldrich), snake venom phosphodiesterase (Sigma Aldrich), and Calf Intestinal Phosphatase (NEB) [2–4,20,51]. The digested samples were separated by analytical HPLC using a Luna C18 reverse-phase column (10 μm, 4.6 mm x 250 mm) (Phenomenex) and as in a previously described protocol [20]. Synthetic 2-methyladenosine and the unmodified mononucleosides were detected by UV absorption at 256 nm, whereas the ^14^C-labeled methyladenosines were detected by radiomatic flow scintillation analyzer (Packard 515TR flow scintillation analyzer; Perkin-Elmer).

## Results

### *In vitro* Methylation of WT tRNAs

**Fig 2:**
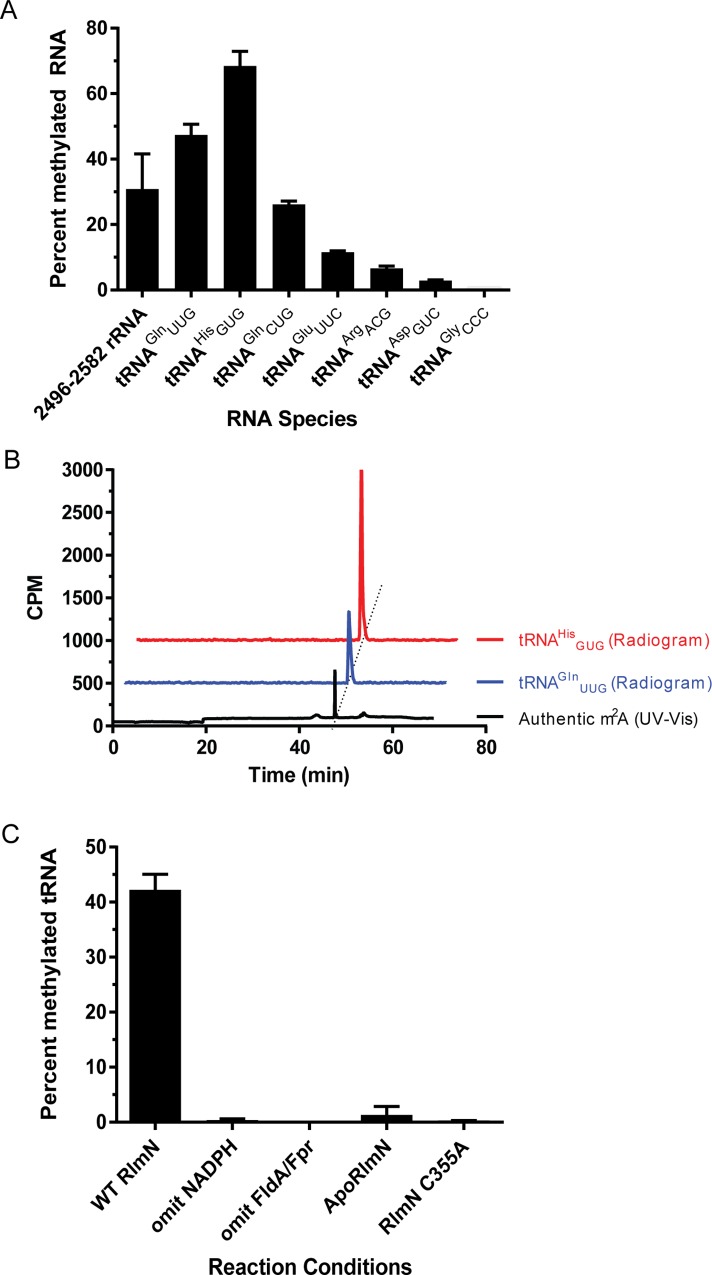
RlmN mediated methylation of *in vitro* transcribed tRNAs. (A) End-point methylation of *in vitro* transcribed RNAs. Bars represent the mean of at least two replicates ± s.d. (B) Radio HPLC analysis of nucleosides obtained by methylation and total RNA digestion of select tRNA substrates. Radioactive signal co-elutes with an authentic m^2^A standard. (C) Evaluation of reaction requirements for methylation of *in vitro* transcribed tRNA^Gln^_UUG_. Bars represent the mean of two replicates ± s.d.

We set out to determine whether we could reconstitute RlmN activity toward *in vitro* transcribed tRNAs that have been previously reported to be methylated at A37 [26]. Our panel included the following tRNAs: tRNA^Arg^_ACG_, tRNA^Asp^_GUC_, tRNA^Gln^_CUG_, tRNA^Gln^_UUG_, tRNA^Glu^_UUC_, and tRNA^His^_GUG_. In addition to the aforementioned substrates, our panel also included tRNA^Gly^_CCC_, which contains an adenosine at A37 but is not known to be modified at this nucleotide by any enzyme, and thus serves as our negative control. Finally, a fragment of 23S rRNA (2496–2582) was selected as a positive control due to its size similarity to tRNA [20]. All RNAs were *in vitro* transcribed, and incubated with RlmN and [^14^C-methyl]-SAM under anaerobic conditions. Following the incubation, RNA was recovered from the reaction and the total radioactivity incorporated into the product was analyzed by liquid scintillation counting (Fig 2A). Unlike tRNA^Gly^_CCC_, all other tRNA transcripts were methylated by *E*. *coli* RlmN *in vitro*. Incorporation of radioactivity into the *in vitro* transcribed tRNAs indicates that RlmN is able to utilize these tRNAs as substrates despite the lack of any additional naturally occurring post-transcriptional modifications. Thus, our *in vitro* reconstituted system recapitulated the *in vivo* findings that tRNA^Arg^_ACG_, tRNA^Asp^_GUC_, tRNA^Gln^_CUG_, tRNA^Gln^_UUG_, tRNA^Glu^_UUC_, and tRNA^His^_GUG_ are all RlmN substrates [26].

To confirm that the observed radioactivity was indeed due to the formation of 2-methyladenosine (m^2^A), we enzymatically performed a total digest of the radiolabeled RNA. The resulting single nucleosides were separated by reverse-phase HPLC and compared to a synthetic standard of m^2^A (Fig 2B). In reactions containing tRNA, we observed formation of a radioactive product that co-eluted with authentic m^2^A. These findings are largely consistent with recent mass-spectrometry based detection of m^2^A obtained by incubation of RlmN with *in vitro* transcribed tRNA [40].

Thus far, the majority of mechanistic studies on RlmN-catalyzed methylation were carried out with rRNA as a substrate [19–21,25]. Here, we investigated if the reaction requirements for tRNA methylation by RlmN are consistent with the previously reported mechanism of rRNA modification by this enzyme. To do so, we omitted various components from the reaction and monitored radioactivity incorporation into tRNA^Gln^_UUG_. Specifically, when reducing equivalents (NADPH or FldA/Fpr) were omitted from the reaction, we did not observe the formation of m^2^A (Fig 2C). Additionally, no product was formed when RlmN devoid of the [4Fe-4S] cluster, (ApoRlmN) was used in the reaction. Finally, no m^2^A formation was detected when Cys118, a residue required for methylation [22,52] was mutated to alanine. In summary, our results demonstrate that RlmN utilizes a similar mechanism to methylate both rRNA and tRNA and preference for certain tRNAs also demonstrates that nucleotide sequence may play a role in influencing methylation by RlmN.

### *In vitro* Methylation of Chimeric Constructs

**Fig 3:**
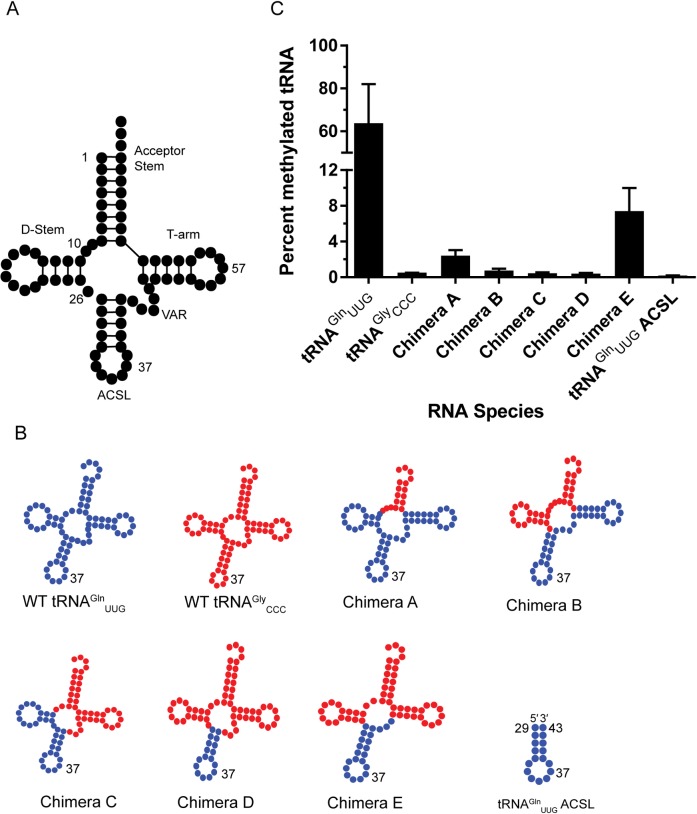
Interrogation of chimeric tRNA substrates. (A) A representative 2D structure of tRNA. (B) *In silico* predicted representation of chimeric tRNA used in this study. Elements derived from substrate tRNA^Gln^_UUG_ are in blue, while those deriving from non-substrate tRNA^Gly^_CCC_ are in red. (C) End-point methylation of chimeric tRNA and tRNA^Gln^_UUG_ ACSL RNA. Bars represent the mean ± s.d. of at least two replicates.

To investigate how structural features of tRNA—namely the D-arm, the T-arm, acceptor stem, ACSL and variable loop region (VAR) (Fig 3A)—impact the ability of tRNA to serve as an RlmN substrate, we sought to generate chimeric constructs. To inform construction of the chimeras, we carried out sequence alignment of *E*. *coli* tRNA sequences obtained from the MODOMICS database (S1 Fig) [23,24,42–44]. In addition to substrate tRNAs, (S1 Fig, blue highlight), our search revealed a number of tRNAs that contain an adenosine at position 37. We selected a subset of these tRNAs, those not known to be modified at position 37, as our non-substrate group (S1 Fig, red highlight). The chimeric constructs combined features of both substrate (tRNA^Gln^_UUG)_) and non-substrate (tRNA^Gly^_CCC_) tRNAs (Fig 3B).

We tested the ability of these chimeric constructs to act as substrates for *E*. *coli* RlmN using an *in vitro* methylation assay. When compared to the WT tRNA^Gln^_UUG_ and tRNA^Gly^_CCC_ (Fig 3C), all chimeric constructs exhibit a drastic reduction in methylation in the endpoint radioactivity incorporation assay (Fig 2C). Radioactivity incorporation was detected for Chimeras A (tRNA^Gly^_CCC_ acceptor stem and tRNA^Gln^_UUG_ body) and E (ACSL and VAR of tRNA^Gln^_UUG_ grafted onto the body of tRNA^Gly^_CCC_), and further verified by RNA digestion and detection of m^2^A (S3 Fig). Chimera B (tRNA^Gly^_CCC_ acceptor and D-arm), Chimera C (tRNA^Gly^_CCC_ acceptor, T-arm, and VAR) and Chimera D (tRNA^Gln^_UUG_ ACSL; tRNA^Gly^_CCC_ body) were poor substrates for methylation, despite appearing well-folded by native gel (S2 Fig). In addition to the chimeras, we tested a 17-nucleotide sequence corresponding to ACSL of tRNA^Gln^_UUG_ ACSL. We concluded that this minimal ACSL alone is not a substrate for methylation by RlmN.

### *In vitro* Methylation of ACSL Point Mutant tRNAs

**Fig 4:**
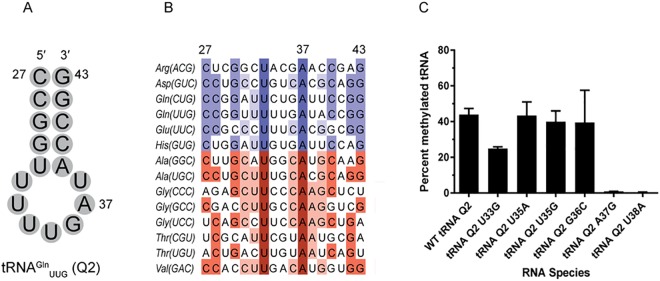
RlmN methylation of ACSL mutants. (A) Sequence of tRNA^Gln^_UUG_ ACSL. Numbers indicate the beginning and end of ACSL, as well as the target adenosine. (B) Alignment of ACSL regions from substrate (blue) and non-substrate (red) *E*. *coli* tRNAs. Darker colors represent a high degree of conservation. Lighter colors indicate that the nucleotide is less conserved. (C) End-point methylation of *in vitro* transcribed tRNA^Gln^_UUG_ point mutants. Bars represent the mean of at least two replicates ± s.d.

**Fig 5:**
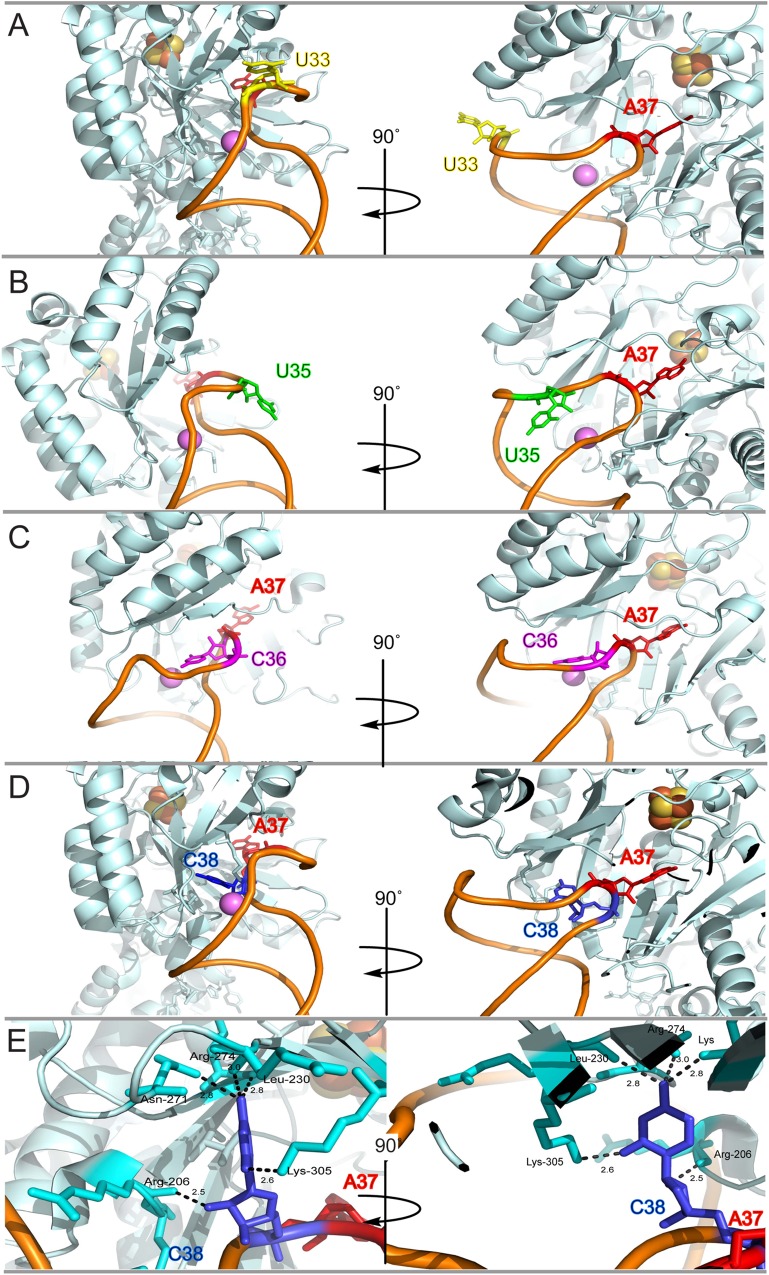
Analysis of ACSL mutations in the context of tRNAGluUUC−RlmN C118A crystal structure [40]. Crystal structure of RlmN C118A in complex with tRNA^Glu^_UUC_ showing the ACSL nucleotides U33 in yellow (A), U35 in green (B), C36 in magenta (C), and C38 in blue (D). Protein contacts between C38 (blue) and RlmN (cyan) are depicted in (E). In all panels, the target adenosine (A37) is depicted in red, the tRNA backbone in orange, the [4Fe-4S] cluster as red and yellow spheres, magnesium as a violet sphere, and RlmN in mint.

To assess the importance of the local RNA sequence on recognition by RlmN, we generated a series of point mutants in the ACSL region of tRNA^Gln^_UUG_ (Fig 4A). We probed nucleotides from positions 33–38 surrounding the substrate adenosine (Fig 5) and tested their ability to serve as RlmN substrates *in vitro*. Mutation at position 33 from uracil to guanosine resulted in a 2-fold decrease in the ability of RlmN to methylate the tRNA substrate. We elected not to mutate position 34, as all possible nucleotides are found at this position in the native substrate tRNAs (Fig 4B). Mutations on nucleotides in positions 35 and 36 had minimal effect on RlmN-mediated methylation, as these mutant tRNAs were methylated at approximately the same level as WT tRNA^Gln^_UUG_. As expected, when A37 was replaced by a G, methylation was abolished. Methylation was also abolished when U38 was mutated to adenosine (Fig 4C).

## Discussion

RlmN, a radical SAM methylating enzyme, has the unusual property of modifying two distinct types of RNA: 23S rRNA and several tRNAs. While recognition of rRNA has been investigated to determine key substrate requirements [20] and characterize aspects of methylation [19–24] relatively little is known about the ability of this enzyme to modify tRNAs. Furthermore, the ability of RlmN to modify only a subset of A37-containing tRNAs further highlights the need for a better understanding of tRNA recognition by this enzyme. Following the initial report of tRNA modification by RlmN [26], only one recent study has investigated tRNA recognition by this enzyme and has provided unprecedented structural insight into how RlmN interacts with tRNA [40]. Here, we examine how RlmN recognizes tRNA *in vitro* by systematic investigation of substrate tRNA modifications, namely through chimeric constructs and point mutants in the ACSL of tRNA. The recently solved crystal structure of RlmN in complex with tRNA^Glu^_UUC_ [40] provides a framework to interpret our results.

In the initial report that identified tRNAs as substrates of RlmN, it was demonstrated that tRNAs tRNA^Arg^_ACG_, tRNA^Asp^_GUC_, tRNA^Gln^_CUG_, tRNA^Gln^_UUG_, tRNA^Glu^_UUC_, and tRNA^His^_GUG_ are *in vivo* substrates for RlmN [26,38,39]. Based on the lack of *in vitro* activity, it was speculated that RlmN acted in late-stage tRNA maturation after other modifications had occurred. Here we show that *in vitro* transcribed tRNAs are indeed substrates for methylation by RlmN (Fig 2), consistent with a recent report [40]. In our end-point methylation assay, we observed that tRNAs tRNA^Gln^_CUG_, tRNA^Gln^_UUG_, tRNA^Glu^_UUC_, and tRNA^His^_GUG_ are better methylation substrates than tRNA^Arg^_ACG_, tRNA^Asp^_GUC_ under the utilized conditions. This difference in substrate preference may result from the nature of the nucleotide in position 29 of tRNA. The Gln, Glu, and His tRNAs contain guanosine in this position, a residue that forms a bidentate hydrogen bond with R206 as shown in the recent crystal structure [40]. In contrast, our alignment of tRNA^Arg^_ACG_ and tRNA^Asp^_GUC_, reveals position 29 to be a C and U, respectively. Overall, these results indicated that *in vitro* transcribed tRNAs are RlmN substrates. Differences in enzyme preparation or conditions of the activity assay may account for the lack of *in vitro* tRNA methylation observed in a previous study [26].

In the context of tRNA methylation, the reaction requirements for substrate modification (Fig 2C) are analogous to those for rRNA methylation [19–22,52]. Analogous to other radical SAM enzymes, the presence of a reductant is required for activity. Additionally, RlmN lacking the iron-sulfur cluster is inactive, consistent with the critical role of the cluster in both C355 methylation and formation of 5'-dA• [20,52,53].

Intrigued by the observation that only a subset of tRNAs containing A37 are modified by RlmN *in vivo*, we generated chimeric constructs combining elements of substrate tRNA^Gln^_UUG_ with that of non-substrate tRNA^Gly^_CCC_, a tRNA that also contains A37 (S1 Fig). The following structural elements of tRNA were exchanged: the D-arm, the T arm, the ACSL, and the VAR. While sequence alignment shows some of these elements are highly conserved between substrates and non-substrates (*e*.*g*. T-arm, S1 Fig) other regions are highly divergent (*e*.*g*. ACSL and VAR, S1 Fig). The generated constructs were analyzed by native gel electrophoresis, which revealed a single band that migrates similar to the wild-type tRNA (S2 Fig). Additionally, the melting temperatures of chimeric constructs are similar to the wild-type tRNAs (S2 Table). Despite appearing well folded by both of these measures, the chimeric tRNAs are poor substrates for RlmN (Fig 3C). Among these poor substrates, Chimera E, containing the ACSL and VAR regions of tRNA Q2 and D and T arms of tRNA^Gly^_CCC_, was methylated 10-fold less than tRNA^Gln^_UUG_ as determined by our radioactivity incorporation assay. Additionally, Chimera A, where the acceptor stem of tRNA^Gln^_UUG_ had been replaced by that of tRNA^Gly^_CCC_, showed a 20-fold drop in methylation by RlmN. Minimal, if any, methylation of Chimeras B, C, and D was observed in these assays.

That the chimeric constructs, even when containing the ACSL region of an efficient WT tRNA substrate, are not efficiently modified suggests that RlmN carefully interrogates multiple features of tRNAs. Modeling with *in silico* prediction software (Fig 3B) [49], reveals that the secondary structures are not dramatically different, however we cannot exclude the possibility that tRNA 3D structure may change in the chimeras. Outside of the ACSL, substrate interactions are mediated by RNA backbone interactions, and slight changes to tertiary structure may prevent adequate recognition by RlmN. The importance of distal regions for recognition is reminiscent of our earlier investigation of rRNA substrate requirements, where helical regions adjacent to substrate A2503 strongly contributed to RlmN methylation [20]. Finally, as expected, the ACSL 17-mer is not modified by RlmN, consistent with the extended recognition of the substrate observed in the crystal structure. This observation contrasts with tRNA recognition by other posttranscriptional modifying enzymes, such as TrmA, an enzyme which efficiently modifies short RNA fragments [8].

To assess the importance of the RNA sequence for methylation by RlmN, we chose to target our efforts to the sequence in the ACSL, as this region shows the most extensive remodeling in the crystal structure. We generated point mutations to the ACSL region of tRNA^Gln^_UUG_ (Q2) and assessed the ability of RlmN to modify these mutants using our *in vitro* methylation assay. When the highly conserved uracil 33 was mutated to guanosine, we observed a modest but reproducible two-fold reduction in methylation. Little to no change was observed for mutations at positions 35 or 36 (Fig 4B). Our observations are consistent with recent structural analysis, which indicates that nucleotides 33 and 35 are not in direct contact with the enzyme. Similarly, the crystal structure shows that position 36 can accommodate G or C (Fig 5); both nucleosides are found in the native tRNAs modified by RlmN. In contrast to these mutations, replacement of the substrate nucleotide (A37G) completely abolishes activity of RlmN, as does replacement of U38 by an adenosine (Fig 4C). In the crystal structure, U38 makes several contacts with the active site residues (Fig 5E) [40]. While these contacts do not appear to be base-specific, it is likely that adenosine in this position would cause significant steric clashing with the protein. Among tRNA substrates, only tRNA^Arg^_ACG_ has an adenosine in position 38 and is among the poorer substrates under our *in vitro* methylation conditions (Fig 2). The remaining tRNA substrates contain a smaller pyrimidine at this position. In contrast, among the tRNAs that have A37 but are not modified by RlmN, five of the eight contain an adenosine at position 38.

In addition to specific nucleotide sequence in the tRNA, it is also interesting to consider the timing of the m^2^A modification. While our *in vitro* results provide evidence that additional modifications are not necessary for methylation by RlmN, the ability of tRNA to be modified by RlmN may be drastically altered by the presence of other modifications. In tRNAs modified by RlmN, there are a number of other ACSL posttranscriptional modifications that occur *in vivo*, including pseudouridine at position 32 by the enzyme RluA [28,31,54] and positions 38, 39 and 40 of tRNA by the enzyme TruA [55], as well as modifications to the wobble position (34) such as queuosine and 5-methylaminomethyl-2-thiouridine. The impact of these modifications on RlmN function is yet to be determined.

In conclusion, we show herein that RlmN is able to modify a number of tRNAs *in vitro*. Using chimeras and point mutations we probed the structure and sequence determinants for methylation by this enzyme. In combination with the recent complementary structural study, our findings have increased understanding of tRNA modification by RlmN, and contribute to the growing understanding of substrate recognition by radical SAM methylating enzymes.

## Supporting Information

S1 Fig Full alignment of substrate and non-substrate RNAs. Substrate tRNAs (blue) and non-substrate tRNAs (red) were aligned in Jalview. Darker shades of color represent a higher degree of conservation at a given position. Less conserved positions are represented by lighter shades of color. The target adenosine is indicated at position 37; sequence elements corresponding to regions of interest (*e*.*g*. ACSL, T-arm) are also denoted. A section of rRNA from Domain V of 23S rRNA is shown for comparison, with the target adenosine (2503) indicated. We excluded tRNAs that do not contain A at position 37, as well as those tRNAs that contain A37 modified by another enzyme.
(TIF)

S2 Fig Native gel analysis of Chimeric tRNA constructs. Chimera tRNA folding was analyzed by 0.5x TBE, 8% polyacrylamide native gel stained with Sybr Gold and visualized on a BioRad ChemiDoc MP Imaging System. Lanes contain the following RNAs: 1. *in vitro* transcribed tRNA^Gln^_UUG_, 2. Chimera A, 3. Chimera B, 4. Chimera C, 5. Chimera D, 6. Chimera E, 7. *in vitro* transcribed tRNA^Gly^_CCC_.
(TIF)

S3 Fig Analysis of methylation of chimeras A and E by radio HPLC. Chimeras A and E were isolated from *in vitro* methylation assay with radioactive SAM, enzymatically digested, and the nucleosides separated by reverse-phase HPLC. The retention time of the methylated nucleoside was compared with an authentic m^2^A standard.
(TIF)

S1 Table Sequences for tRNA truncations and Chimera tRNA constructs. Chimera tRNAs were generated by overlap extension PCR. Sequences for tRNA^Gln^_UUG_ (“substrate”) and tRNA^Gly^_CCC_ (“non-substrate”) were obtained from the MODOMICS RNA database. Sequence elements originating from the substrate are in UPPERCASE; those originating from the non-substrate are lowercase. The target adenosine is denoted in bold underline.
(XLSX)

S2 Table Melting temperature (T_m_) for select chimera tRNA constructs. Melting temperature was calculated as the inflection point (d^2^A/dT^2^) of the absorbance with respect to temperature. All values represent the mean ± s.d. of a minimum of three replicates.
(XLSX)
