# Supplementary figures and images for: Determinants of tRNA Recognition by the Radical SAM Enzyme RlmN
Source: PLoS One. 2016 Nov 30;11(11):e0167298. doi: 10.1371/journal.pone.0167298 (PMC5130265; doi:10.1371/journal.pone.0167298)

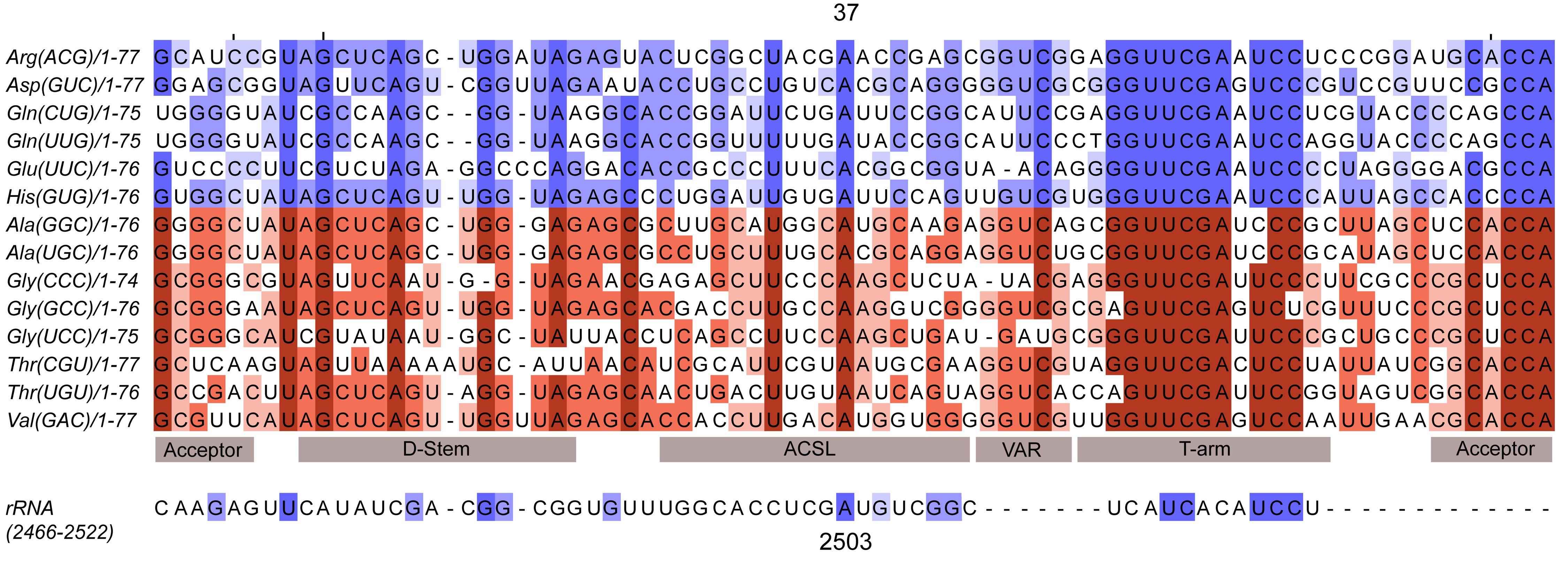

Supplement: S1 Fig — Substrate tRNAs (blue) and non-substrate tRNAs (red) were aligned in Jalview. Darker shades of color represent a higher degree of conservation at a given position. Less conserved positions are represented by lighter shades of color. The target adenosine is indicated at position 37; sequence elements corresponding to regions of interest (e.g. ACSL, T-arm) are also denoted. A section of rRNA from Domain V of 23S rRNA is shown for comparison, with the target adenosine (2503) indicated. We excluded tRNAs that do not contain A at position 37, as well as those tRNAs that contain A37 modified by another enzyme. (TIF) [file pone.0167298.s001.tif]

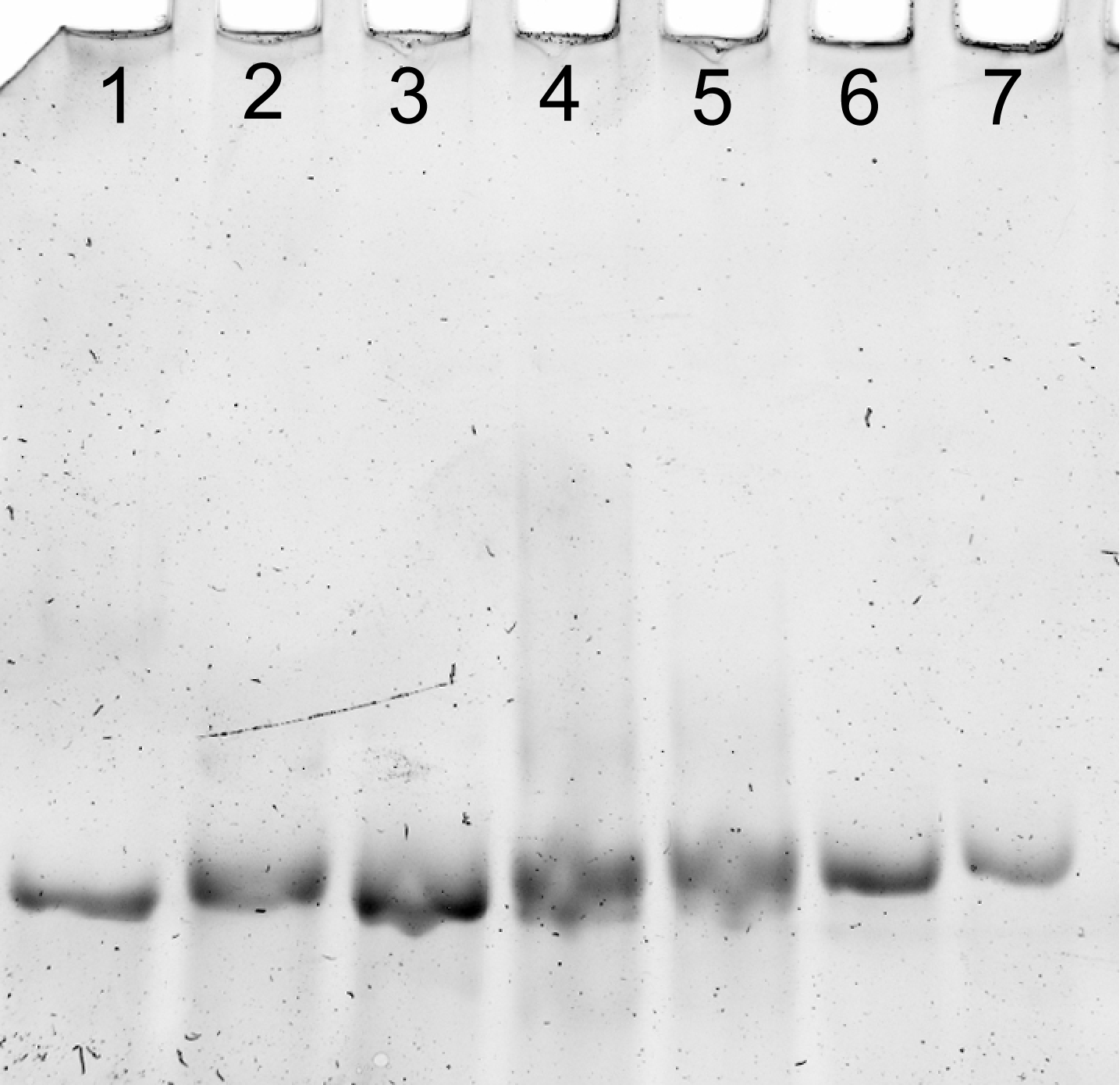

Supplement: S2 Fig — Chimera tRNA folding was analyzed by 0.5x TBE, 8% polyacrylamide native gel stained with Sybr Gold and visualized on a BioRad ChemiDoc MP Imaging System. Lanes contain the following RNAs: 1. in vitro transcribed tRNAGlnUUG, 2. Chimera A, 3. Chimera B, 4. Chimera C, 5. Chimera D, 6. Chimera E, 7. in vitro transcribed tRNAGlyCCC. (TIF) [file pone.0167298.s002.tif]

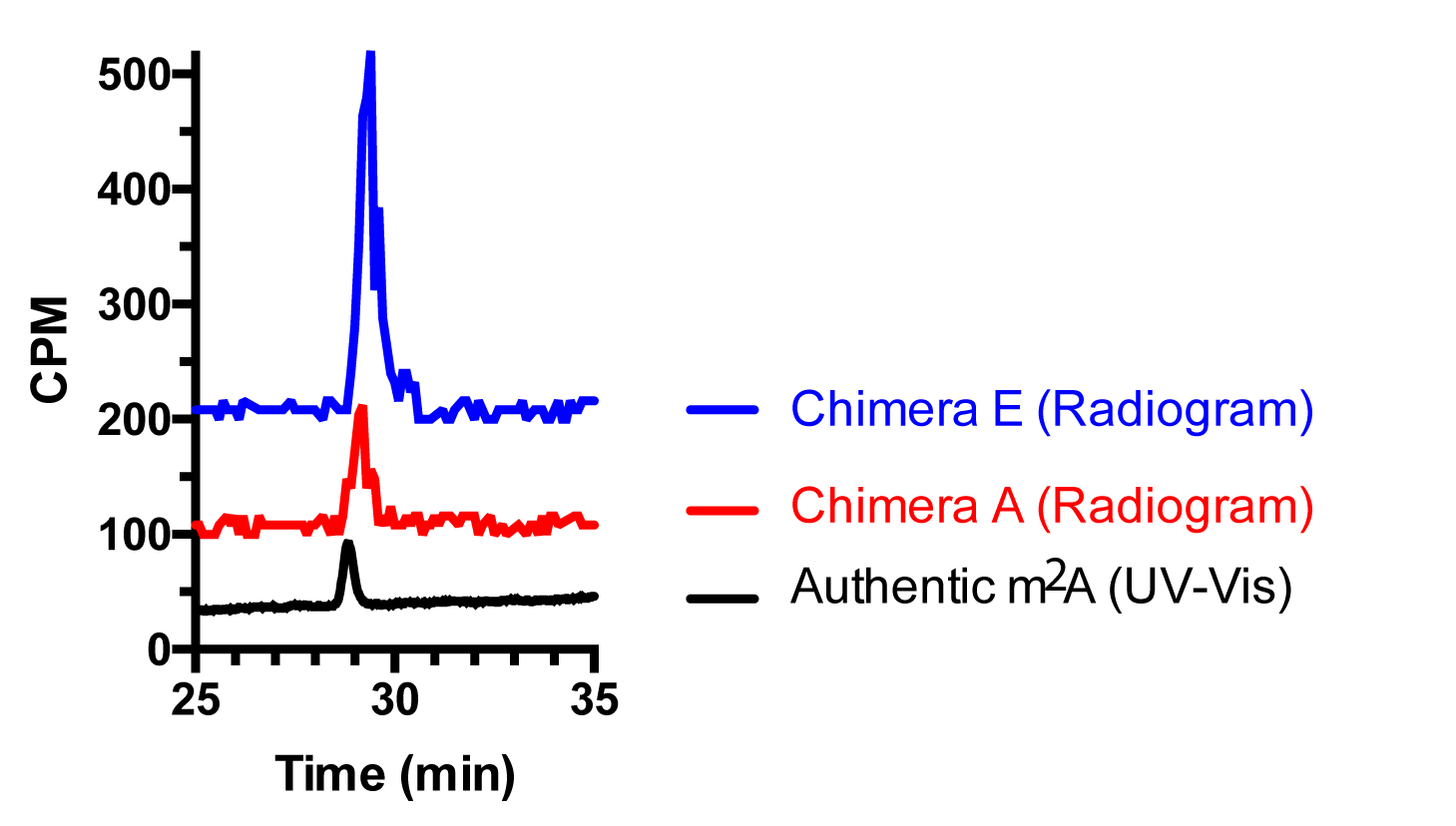

Supplement: S3 Fig — Chimeras A and E were isolated from in vitro methylation assay with radioactive SAM, enzymatically digested, and the nucleosides separated by reverse-phase HPLC. The retention time of the methylated nucleoside was compared with an authentic m2A standard. (TIF) [file pone.0167298.s003.tif]
